# Prognostic value of serum cystatin C concentration in dogs with myxomatous mitral valve disease

**DOI:** 10.1111/jvim.16669

**Published:** 2023-02-27

**Authors:** Naoki Iwasa, Rie Kumazawa, Saki Nomura, Mamu Shimizu, Munetaka Iwata, Mayuka Hara, Mifumi Kawabe, Yui Kobatake, Satoshi Takashima, Naohito Nishii

**Affiliations:** ^1^ Hashima Animal Hospital Gifu Japan; ^2^ Joint Department of Veterinary Medicine Faculty of Applied Biological Sciences Gifu Japan; ^3^ Animal Medical Center Gifu University Gifu Japan

**Keywords:** cystatin C, heart failure, myxomatous mitral valve disease, renal function

## Abstract

**Background:**

Impaired renal function is 1 of the poor prognostic factors in dogs with myxomatous mitral valve disease (MMVD). However, the value of cystatin C (Cys‐C), a marker of renal function, as a prognostic marker for MMVD in dogs has not yet been explored.

**Objective:**

This study aims to investigate the prognostic value of Cys‐C in dogs with MMVD.

**Animals:**

Fifty client‐owned small‐breed dogs with MMVD were included in this study.

**Methods:**

This is a retrospective, cross‐sectional study. The prognostic value of serum Cys‐C concentration was assessed using univariable and multivariable Cox hazard regression analyses. Kaplan‐Meier survival curves for MMVD‐specific survival in dogs stratified into high and low Cys‐C groups were generated and analyzed using the log‐rank test.

**Results:**

Serum Cys‐C concentrations were significantly associated with MMVD‐related death (*P* < .01) in both univariable (hazard ratio [HR], 5.086; 95% confidence interval [CI], 1.950‐13.270) and multivariable Cox hazard regression analysis (HR, 4.657; 95% CI, 1.767‐12.270). The high Cys‐C group (n = 14) had a significantly shorter MMVD‐specific survival time than the low Cys‐C group (n = 36; *P* < .01). In dogs with normal blood creatinine concentrations, the high Cys‐C group (n = 10) had a significantly shorter MMVD‐specific survival time than the low Cys‐C group (n = 36; *P* < .01).

**Conclusions and Clinical Importance:**

High serum Cys‐C concentrations were associated with a worse prognosis of MMVD. Furthermore, serum Cys‐C could be a predictor of MMVD prognosis even in dogs with normal blood creatinine concentration.

**Abbreviations**
ACE: angiotensin converting enzyme
ACVIM: American College of Veterinary Internal Medicine
ANP: atrial natriuretic peptide
BUN: blood urea nitrogen
Cr: creatinine
Cys‐C: cystatin C
GFR: glomerular filtration rate
LA/AO: left atrial‐to‐aortic ratio
LVIDDN: left ventricular end‐diastolic internal diameter
MMVD: myxomatous mitral valve disease
UPC: urea protein/creatinine
USG: urinary specific gravity
VHS: vertebral heart score
VLAS: vertebral left atrial size

## 1 INTRODUCTION

Myxomatous mitral valve disease (MMVD) is the most common heart disease in small‐breed dogs and can result in death because of pulmonary edema, syncope, and dyspnea caused by left ventricular volume overload.^1^ The severity of MMVD is classified by the American College of Veterinary Internal Medicine (ACVIM) stages. The ACVIM stage was related to the prognosis of dogs with MMVD, and the higher the stage, the worse the prognosis.^1^ Renal function is 1 of the important prognostic factors for dogs with MMVD.^1^, ^2^ Renal dysfunction could be a negative prognostic factor in dogs with MMVD.^2^ The most common marker of renal function is serum creatinine (Cr), and the survival time of dogs with MMVD and high serum Cr concentrations is shorter than that of dogs with MMVD and low serum Cr concentrations.^2^ However, serum Cr concentrations do not increase until the glomerular filtration rate (GFR) decreases by 75%.^3^ Furthermore, their serum Cr concentrations tend to remain normal because small‐breed dogs have less skeletal muscle mass.^4^ In addition, dogs with severe MMVD often develop cardiac cachexia, which can lead to lower serum Cr. Therefore, more sensitive markers that are independent of nonrenal factors and can detect renal dysfunction and prognosis earlier than serum Cr in dogs with MMVD are needed.

Serum cystatin C (Cys‐C) is used in dogs as a GFR biomarker.^5^, ^6^ In humans, serum Cys‐C is a better GFR marker for renal disease than serum Cr.^7^, ^8^, ^9^, ^10^ Serum Cys‐C concentrations are significantly higher in dogs with renal failure than in clinically healthy individuals.^11^, ^12^ Despite that it is only useful in small‐breed dogs, serum Cys‐C is a better GFR marker than serum Cr.^13^, ^14^ Thus, serum Cys‐C could be a promising sensitive renal marker in small‐breed dogs. In humans, serum Cys‐C concentrations are linked with prognosis not only in patients with renal insufficiency but also in patients with heart failure. Higher serum Cys‐C concentrations have been detected in human patients with heart disease and have been linked to heart failure and a poor prognosis, particularly in those with coronary artery disease.^15^, ^16^, ^17^, ^18^, ^19^, ^20^ Furthermore, serum Cys‐C concentrations in human patients detect a poor prognosis of heart disease earlier than serum Cr concentrations.^17^ However, the prognostic value of Cys‐C in dogs with heart disease has not yet been reported. The present study aims to evaluate the prognostic value of serum Cys‐C concentrations in dogs with MMVD.

## 2 MATERIALS AND METHODS

### 2.1 Case selection

This is a retrospective cross‐sectional study. The data were acquired from the medical records of dogs diagnosed with MMVD at a primary care veterinary hospital between February 2015 and April 2021. Dogs weighting ≥15 kg were excluded from this study because serum Cys‐C concentration is an inferior renal marker in larger breed dogs.^13^ Of the dogs included, those with missing data required for this study were excluded from the study. Dogs who had received oral administration of prednisolone within the previous month were excluded because oral administration of prednisone increases serum Cys‐C concentrations.^21^ In addition, dogs with atrial flutter, fibrillation, other concomitant cardiac (eg, cardiomyopathy or infective endocarditis) and systemic diseases, as well as dogs that had mitral valve repair surgery, were excluded. Data on the cause of death, survival period, medication history, clinical manifestations, body weight, age, sex, breed, serum Cys‐C concentration, blood urea nitrogen (BUN) concentration, blood Cr concentration, urinary specific gravity (USG), urea protein/Cr ratio (UPC), plasma atrial natriuretic peptide (ANP) concentration, vertebral heart score (VHS), vertebral left atrial size (VLAS), left atrial‐to‐aortic ratio (LA/AO), and left ventricular end‐diastolic internal diameter (LVIDDN) were extracted from the medical record. Information about feeding status was not available. According to the American College of Veterinary Internal Medicine (ACVIM) consensus guidelines, the MMVD stage was classified as B1, B2, C, or D.^1^ Survival was followed up, and dates of MMVD‐related deaths and deaths from other causes were recorded. MMVD‐related deaths were defined as deaths occurring as a result of progression of MMVD evidenced by 1 or more of pulmonary edema, syncope, or dyspnea. This study was approved by the local ethics committee for animal clinical research (approval no. E22002). Informed consent by dog owners was waived because of the study's retrospective nature. All dog owners were given the option to opt out of the present study, which was conveyed via the animal hospital's bulletin board.

### 2.2 Chest radiography

The VHS^1^, ^22^, ^23^ and VLAS^1^, ^24^ were measured using lateral thoracic radiography, as previously described.

### 2.3 Echocardiography

The LA/AO ratio and LVIDDN were measured using echocardiographic images. M‐mode, Doppler, and 2‐dimensional (2D) echocardiography was performed by a veterinarian using an ultrasound unit (Noblus, FUJIFILM Healthcare, Tokyo, Japan) with a 2.0‐ to 9.0‐MHz sector probe (S‐31 Probe, FUJIFILM Healthcare). During the examination, dogs were restrained on the right lateral recumbency. The LA/AO ratio was calculated from the right parasternal short‐axis 2D view on the first frame after the aortic valve was closed.^1^, ^25^, ^26^ The LVIDDN was calculated as follows: $\text{left ventricular dimension}\;\mathrm{end}\;\text{diastolic diameter}\;\left(\mathrm{cm}\right)/\text{weight}\;{\left(\mathrm{kg}\right)}^{0.294}$.^1^, ^27^

### 2.4 Blood biochemistry

Data on blood biochemistry was obtained during the initial visit. Blood was collected from the cephalic vein and delivered into plain, heparin, and aprotinin tubes. After allowing the samples in plain tubes to clot, the serum was separated by centrifugation at 5000 rpm for 4 minutes at room temperature. Plasma was separated by centrifuging heparin tubes at 5000 rpm for 4 minutes at room temperature. Aprotinin tubes were centrifuged at 3000 rpm for 10 minutes at 4°C to separate aprotinin plasma. Serum Cys‐C concentrations were measured using a latex immunoturbidimetric assay designed for human use (Iatro Cys‐C, LSI Medience, Tokyo, Japan) on an automatic analyzer (JCA‐BM 6070, JEOL, Tokyo, Japan) that had previously been validated in dogs.^14^ Serum was used to measure BUN and Cr concentrations, while plasma was used in 2 dogs. The BUN and Cr concentrations were measured using enzyme assays (vBUN‐P and vCre‐P, Fujifilm, Tokyo, Japan, respectively) on an automatic analyzer (DRI‐CHEM NX500V, FUJIFILM Healthcare Co, Ltd). Plasma ANP concentrations in aprotinin plasma were measured using a chemiluminescent enzyme immunoassay (Determiner CL ANP, Hitachi Chemical Diagnostics Systems, Tokyo, Japan) on a fully automated chemiluminescence system (CL‐JACK RK, Hitachi Chemical Diagnostics Systems) that had previously been validated in dogs.^28^

### 2.5 Urinalysis

Urinary specific gravity was measured using a clinical refractometer (MASTER‐URC, ATAGO, Tokyo, Japan). Urinary protein (Micro TP‐AR, Fujifilm WAKO Pure Chemical, Osaka, Japan) and Cr concentrations (L‐type Wako Cre‐M, Fujifilm WAKO Pure Chemical) were measured using a colorimetric assay on an automatic analyzer (JCA‐BM 6070, JEOL), and the UPC ratio was then calculated.

### 2.6 Statistical analysis

Statistical analyses were performed using the R software (The R Foundation for Statistical Computing, version 3.0.2) in combination with the EZR software.^29^ Serum Cys‐C concentrations were compared among various ACVIM stages using the Kruskal‐Wallis test. The cutoff values for Cox proportional hazard regression analysis were determined using the manufacturer's reference value of BUN, blood Cr, and Cys‐C, as well as the median for other variables. The prognostic value of age, body weight, serum Cys‐C concentration, BUN concentration, blood Cr concentration, USG, UPC, ACVIM stage, ANP concentration, VHS, VLAS, LA/AO ratio, and LVIDDN was assessed using a univariable Cox proportional hazard regression model. Furthermore, multivariable Cox proportional hazard regression analysis was performed with serum Cys‐C concentration and age as possible confounders, as serum Cys‐C concentrations were higher in older dogs.^30^, ^31^ Proportional hazard assumption was checked graphically by complementary log‐log plot. The Mann‐Whitney *U*‐test was used to compare the values of variables between groups with Cys‐C higher and equal or lower than the upper reference limit (0.4 mg/L). Kaplan‐Meier survival curves for MMVD or all‐cause survival rates in dogs were generated and analyzed using the log‐rank test. A *P* value of <.05 was considered statistically significant.

## 3 RESULTS

### 3.1 Dogs

**FIGURE 1:**
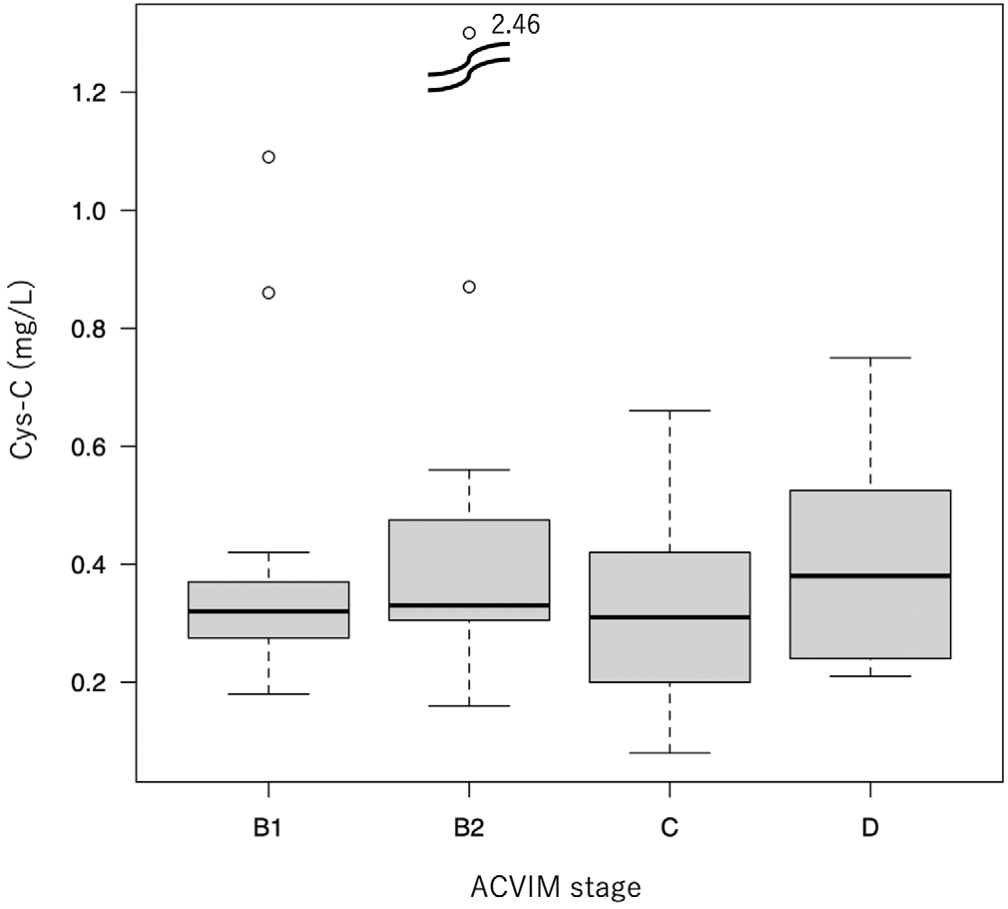
Box and whisker plot of serum Cys‐C concentrations in dogs with various myxomatous mitral valve disease stages. Fifteen dogs were on stage B1, 11 dogs on stage B2, 16 dogs on stage C, and 8 dogs on stage D. The serum Cys‐C concentrations were compared between groups using the Kruskal‐Wallis test, and they did not differ significantly between ACVIM stages.

During the study period, 88 dogs were diagnosed with MMVD, with 64 having complete data for use in this study. Furthermore, 10 dogs with cardiac disease other than MMVD, 2 dogs who had received oral administration of prednisolone within the previous month, and 2 dogs who had mitral valve repair surgery were excluded from this study. Finally, 50 dogs with MMVD were included in the study: 9 males, 19 neutered males, 4 females, and 18 spayed females. Dog breeds included 20 Chihuahua, 9 Cavalier King Charles Spaniel, 7 Miniature Schnauzer, 5 Pomeranian, 4 mixed breeds, 1 Shetland Sheepdog, 1 Shih Tzu, 1 Yorkshire Terrier, 1 Papillon, and 1 Beagle. Other dogs' characteristics in this study are shown in Table 1. According to the ACVIM consensus guidelines for MMVD, the included dogs were classified into 4 stages, with 15, 11, 16, and 8 dogs in stages B1, B2, C, and D, respectively. Serum Cys‐C concentrations did not differ significantly between ACVIM stages (Figure 1). Dogs in stages B1 and B2 were asymptomatic and were not treated for heart disease. All dogs diagnosed with ACVIM stage B2 were subsequently administered pimobendan. However, 16 dogs in stage C had clinical signs (eg, cough (10/16), exercise intolerance (10/16), dyspnea (4/16), or tachypnea/labored respiration with pulmonary edema (2/16)) or cyanosis (1/16) and were treated with furosemide (8/16), angiotensin‐converting enzyme (ACE) inhibitors (2/16), spironolactone (1/16), or pimobendan (10/16). Furthermore, 8 dogs in stage D had clinical signs, for example, cough (6/8), exercise intolerance (4/8), dyspnea (2/8), or tachypnea/labored respiration with pulmonary edema (2/8), and were treated with furosemide (8/8), pimobendan (8/8), spironolactone (7/8), Sildenafil (3/8), or ACE inhibitors (1/8).

**TABLE 1:** Variables of the 50 dogs included in this study.

		n = 50	
Parameters		Median	Range
Age	(year)	12.0	3.0‐15.0
Body weight	(kg)	5.13	2.08‐14.04
Cys‐C	(mg/L)	0.32	0.08‐2.46
BUN	(mg/dL)	21.0	8.0‐140.0
Cr	(mg/dL)	0.78	0.3‐3.4
USG		1.025	1.006‐1.040
UPC		0.185	0.06‐2.46
ANP	(pg/mL)	154.5	35.5‐2483.3
VHS		10.8	9.1‐16.0
VLAS		2.2	1.4‐3.9
LA/AO		1.64	1.14‐3.29
LVIDDN		1.74	1.29‐2.97

Abbreviations: ANP, atrial natriuretic peptide; BUN, blood urea nitrogen; Cr, creatinine; Cys‐C, cystatin C; LA/AO, left atrial‐to‐aortic ratio; LVIDDN, left ventricular end‐diastolic internal diameter; MMVD, myxomatous mitral valve disease; UPC, urea protein/creatinine; USG, urinary specific gravity; VHS, vertebral heart score; VLAS, vertebral left atrial size.

### 3.2 Serum Cys‐C concentration and prognosis of MMVD

The median follow‐up period was 358 days (range, 6‐1901). During the follow‐up period, 21 of the 50 dogs expired from MMVD‐related causes (n = 21), with 5 of 15 dogs at stage B1, 2 of 11 dogs at stage B2, 7 of 16 dogs at stage C, and 7 of 8 dogs at stage D dying from MMVD‐related causes. However, 8 of the 50 dogs died from other causes, including renal disease (n = 2), malignant tumors (n = 4), neurologic disease (n = 1), and hepatic failure (n = 1).

Univariable Cox hazard regression analysis revealed that serum Cys‐C, BUN, ANP, LVIDDN (*P* < .01), blood Cr, and ACVIM stage D (*P* < .05) were all significantly associated with MMVD‐related death (Table 2). Furthermore, multivariable Cox hazard regression analysis with age as a confounding factor revealed that high serum Cys‐C concentration (*P* < .01) remained significantly associated with MMVD‐related death (Table 3).

**TABLE 2:** Results of univariable Cox proportional hazards analysis on MMVD‐related deaths.

Variables	Category		n	Hazard ratio	95% confidence interval	*P*
Age	≤12	(year)	31	1		
	>12	(year)	19	2.148	0.907‐5.087	.08
Body weight	≤5.1	(kg)	25	1		
	>5.1	(kg)	25	102	0.425‐2.447	.96
Cys‐C	≤0.4	(mg/L)	36	1		
	>0.4	(mg/L)	14	5.086	1.950‐13.270	<.01
BUN	≤29.2	(mg/dL)	35	1		
	>29.2	(mg/dL)	15	4.132	1.680‐10.160	<.01
Cr	≤1.4	(mg/dL)	46	1		
	>1.4	(mg/dL)	4	3.975	1.088‐14.520	<.05
USG	≤1.035		25	1		
	>1.035		25	0.743	0.313‐1.765	.51
UPC	≤0.19		26	1		
	>0.19		24	1.741	0.7201‐4.211	.22
ACVIM stage	B1		15	1		
	B2		11	0.496	0.096‐2.560	.41
	C		16	1.637	0.508‐5.280	.41
	D		8	3.462	1.082‐11.080	<.05
ANP	≤154.5	(pg/mL)	25	1		
	>154.5	(pg/mL)	25	1.001	1.000‐1.002	<.01
VHS	≤10.8		26	1		
	>10.8		24	1.516	0.635‐3.616	.35
VLAS	≤2.2		26	1		
	>2.2		24	1.8	0.754‐4.295	.19
LA/AO	≤1.64		26	1		
	>1.64		24	1.334	0.557‐3.192	.52
LVIDDN	≤1.74		26	1		
	>1.74		24	4.669	1.752‐12.44	<.01

Abbreviations: ANP, atrial natriuretic peptide; ACVIM, American College of Veterinary Internal Medicine; BUN, blood urea nitrogen; Cr, creatinine; Cys‐C, cystatin C; LA/AO, left atrial‐to‐aortic ratio; LVIDDN, left ventricular end‐diastolic internal diameter; MMVD, myxomatous mitral valve disease; UPC, urea protein/creatinine; USG, urinary specific gravity; VHS, vertebral heart score; VLAS, vertebral left atrial size.

**TABLE 3:** Results of multivariable Cox proportional hazards analysis on MMVD‐related deaths.

Variables	Category		Hazard ratio	95% confidence interval	*P*
Cys‐C	>0.4	(mg/L)	4.657	1.767‐12.270	<.01
Age	>12	(year)	1.827	0.762‐4.380	.18

Abbreviation: Cys‐C, cystatin C.

### 3.3 Serum Cys‐C concentration and MMVD‐related death

**FIGURE 2:**
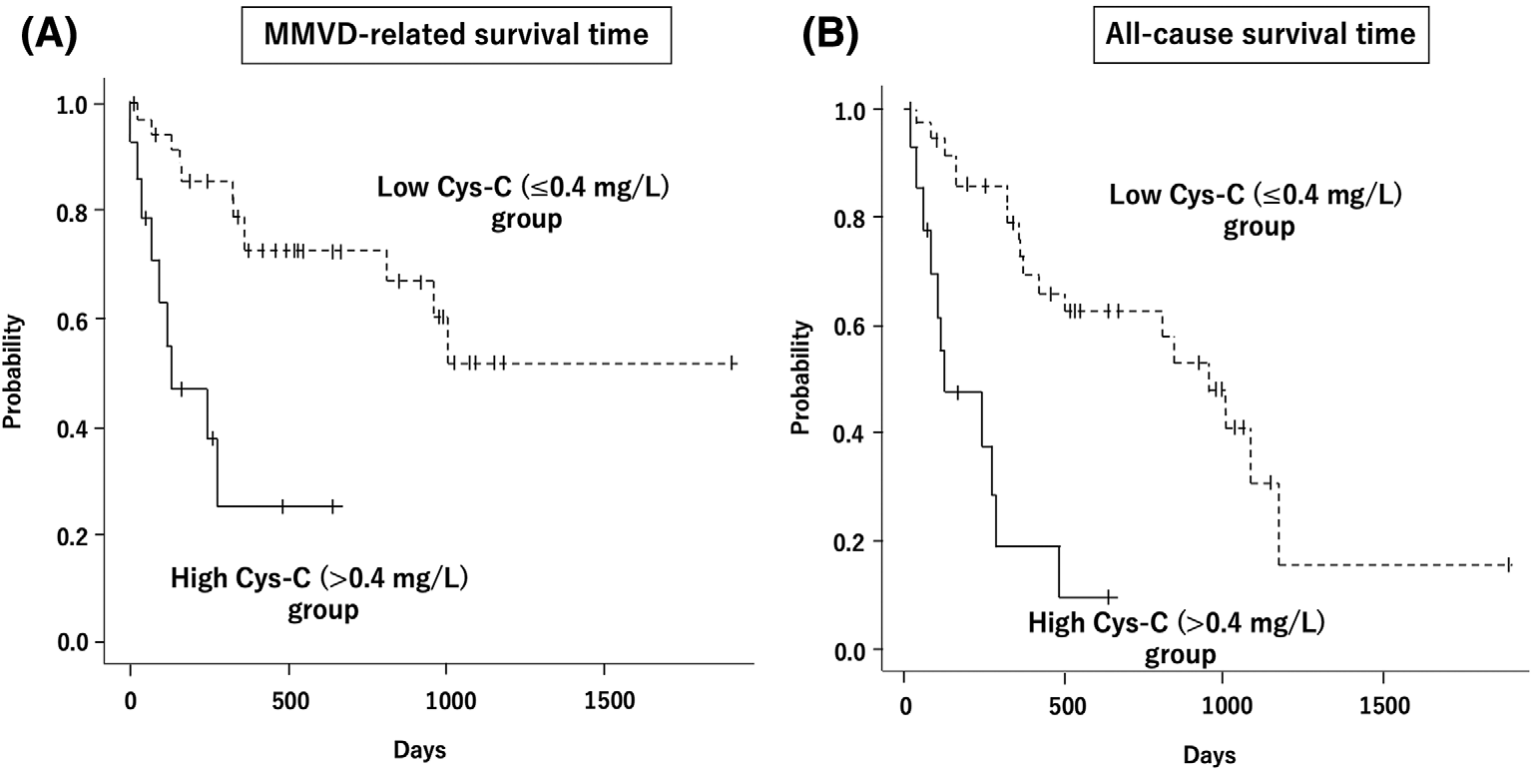
Kaplan‐Meier survival curves in the high and low Cys‐C groups. (A) MMVD‐related survival time and (B) all‐cause survival time. The high Cys‐C group (>0.4 mg/L, n = 14) had significantly shorter MMVD‐specific and all‐cause survival times than the low Cys‐C group (≤0.4 mg/L, n = 36; *P* < .01). Vertical lines censored dogs, solid line high Cys‐C group, and dashed line low Cys‐C group.

MMVD‐specific survival time was analyzed in high (>0.4 mg/L) and low (≤0.4 mg/L) Cys‐C groups. Table 4 shows the variables in the high (n = 14) and low (n = 36) Cys‐C groups. The high Cys‐C group had significantly higher serum Cys‐C concentrations, BUN concentrations, blood Cr concentrations (all *P* < .01), age, UPC, and LVIDDN (*P* < .05) than the low Cys‐C group. MMVD‐related death occurred in 9 of the 14 dogs in the high Cys‐C group and 12 of the 36 dogs in the low Cys‐C group. The high Cys‐C group had significantly shorter MMVD‐specific and all‐cause survival times than the low Cys‐C group (*P* < .01; Figure 2).

**TABLE 4:** Comparison of variables between the low and high Cys‐C groups.

		Low Cys‐C (≤0.4 mg/L)		High Cys‐C (>0.4 mg/L)		
		n = 36		n = 14		
Parameters		Median	Range	Median	Range	*P*
Age	(year)	11.0	3.0‐15.0	12.5	10.0‐15.0	<.05
Body weight	(kg)	5.45	2.08‐13.46	3.99	2.65‐14.04	.64
Cys‐C	(mg/L)	0.30	0.08‐0.39	0.62	0.42‐2.46	<.01
BUN	(mg/dL)	17.5	8.0‐36.1	33.9	17.1‐140.0	<.01
Cr	(mg/dL)	0.7	0.3‐1.2	1.2	0.6‐3.4	<.01
USG		1.029	1.014‐1.040	1.018	1.006‐1.038	.11
UPC		0.16	0.06‐0.40	0.25	0.1‐2.46	<.05
ANP	(pg/mL)	124.7	40‐1052.4	157.0	35.5‐2483.3	.63
VHS		10.8	9.1‐13.1	10.9	9.7‐16.0	.61
VLAS		2.2	1.4‐3.9	2.7	1.8‐3.8	.15
LA/AO		1.65	1.15‐3.29	1.61	1.14‐2.95	.55
LVIDDN		1.72	1.29‐2.97	2.13	1.29‐2.87	<.05

Abbreviations: ANP, atrial natriuretic peptide; BUN, blood urea nitrogen; Cr, creatinine; Cys‐C, cystatin C; LA/AO, left atrial‐to‐aortic ratio; LVIDDN, left ventricular end‐diastolic internal diameter; UPC, urea protein/creatinine; USG, urinary specific gravity; VHS, vertebral heart score; VLAS, vertebral left atrial size.

### 3.4 Survival analysis in dogs with normal blood Cr concentrations

**FIGURE 3:**
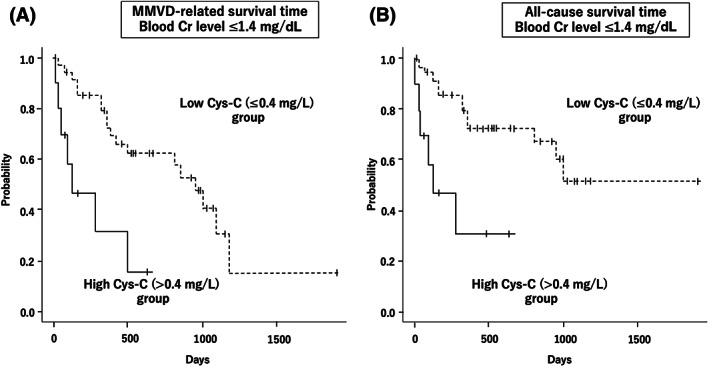
Kaplan‐Meier survival curves in dogs with normal blood creatinine concentrations. (A) MMVD‐related survival time and (B) all‐cause survival time. Even in dogs with normal blood creatinine concentrations, the high Cys‐C group (>0.4 mg/L, n = 10) had significantly shorter MMVD‐specific and all‐cause survival times than the low Cys‐C group (≤0.4 mg/L, n = 36; *P* < .01). Vertical lines censored dogs, solid line high Cys‐C group, and dashed line low Cys‐C group.

A subgroup analysis was performed on 46 dogs with normal blood Cr concentrations (≤1.4 mg/dL), with 10 dogs assigned to the high Cys‐C (>0.4 mg/L) group and 36 dogs assigned to the low Cys‐C (≤0.4 mg/L) group. Six of the 10 dogs in the high Cys‐C group and 12 of the 36 dogs in the low Cys‐C group expired from MMVD‐related causes. Even in dogs with normal blood Cr concentrations, the high Cys‐C group had significantly shorter MMVD‐specific and all‐cause survival times than the low Cys‐C group (*P* < .01; Figure 3).

## 4 DISCUSSION

In this study, the high Cys‐C group had a shorter MMVD‐specific survival time than the low Cys‐C group. Thus, high serum Cys‐C concentrations in dogs with MMVD are suggested to be associated with MMVD‐related deaths and poor prognosis for MMVD. This study establishes that a high serum Cys‐C concentration is a negative prognostic factor for MMVD in dogs.

Serum Cys‐C is a marker of renal function that reflects GFR.^13^ Therefore, the current findings imply that impaired renal function might be associated with MMVD progression. Previous reports have also indicated that dogs with MMVD and chronic kidney disease (CKD) have a worse prognosis than those without CKD.^2^ Cardiovascular‐renal disease (CvRD), a condition in which both cardiac and renal dysfunction negatively affect each other,^2^, ^32^ is caused by sympathetic activation, renin‐angiotensin‐aldosterone system activation, hypertension, and GFR reduction.^32^ CvRD might have rapidly worsened MMVD in dogs with high Cys‐C concentrations, resulting in a poor prognosis for MMVD. However, the present study was unable to determine whether elevated Cys‐C concentrations are linked to CvRD. It is unknown whether Cys‐C is associated with causes of death other than MMVD and kidney disease. However, only 6 dogs died from other causes and in these 6 dogs, serum Cys‐C concentrations were low (data not shown). Therefore, it is unlikely that serum Cys‐C concentrations are associated with causes of death other than MMVD and kidney disease.

In this study, high serum Cys‐C concentrations tended to worsen MMVD prognosis even in dogs with normal blood Cr concentrations. These findings are consistent with those reported in human cardiac patients, where high serum Cys‐C concentrations were associated with a poor prognosis of cardiac disease even with normal serum Cr concentrations.^17^, ^33^ In small‐breed dogs, serum Cys‐C is more sensitive than serum Cr in identifying reduced GFR.^13^ In dogs with normal Cr but high Cys‐C, renal function was impaired, which might have contributed to a worse prognosis of MMVD. Thus, even in dogs with normal Cr, it could be useful to measure Cys‐C to evaluate the prognosis of MMVD.

There is a correlation between MMVD severity and the International Renal Interest Society stage of CKD in dogs.^2^ In addition, the higher the MMVD stage, the higher the serum Cr and Cys‐C concentrations in dogs.^34^ However, in this study, no significant difference in the serum Cys‐C concentrations was noted between the ACVIM stages of MMVD, and dogs in the high Cys‐C group did not necessarily have advanced MMVD stage. The LVIDDN was significantly higher in the high Cys‐C group than in the low Cys‐C group, without significant differences in other MMVD‐related factors (eg, VHS, VLAS, LA/AO ratio, and plasma ANP concentration). These findings indicate that dogs with high serum Cys‐C concentrations did not necessarily have an advanced MMVD at the time of study enrolment. In humans, serum Cys‐C concentration does not correlate with cardiac disease‐related factors and was an independent prognostic factor for cardiac disease.^16^, ^17^ Serum Cys‐C, like in humans, has been suggested to be an independent predictor of MMVD prognosis in dogs.

In this study, the high Cys‐C group was significantly older than the low Cys‐C group. Previous studies have discovered that serum Cys‐C concentrations correlate with age,^30^, ^31^, ^35^, ^36^, ^37^ and that aging reduces renal function while increasing Cys‐C concentrations in humans.^35^, ^36^, ^37^ Moreover, Cys‐C concentrations are higher in elderly dogs than in younger ones.^30^, ^31^ Therefore, in this study, considering the possibility that older age might have contributed to the relationship between high Cys‐C concentrations and a poorer prognosis of MMVD was necessary. Multivariable analysis using age as a confounding factor revealed that serum Cys‐C concentration remained significantly associated with MMVD‐related death. These findings imply that the poor MMVD prognosis in dogs with elevated serum Cys‐C concentrations was independent of age.

This study has some limitations. First, a limited number of samples exist. However, the prognosis between the high and low Cys‐C groups differed markedly. Therefore, the present study's sample size was sufficient to demonstrate the difference. Second, data on GFR measurements were missing because of the retrospective nature of the study. If GFR had been assessed in this study, it might have demonstrated that the poor prognosis caused by elevated serum Cys‐C concentrations was attributed to impaired renal function. Third, data on body condition scores and feeding status were missing. Obesity might affect serum Cys‐C concentrations.^38^ Although Cys‐C is unaffected by diet in cats^39^ and humans,^40^, ^41^ it can be affected by diet in humans.^42^, ^43^ Therefore, the possibility that the degree of obesity or the interval between feedings altered serum Cys‐C concentrations in the present study cannot be ruled out.

In conclusion, the present study demonstrated that serum Cys‐C was a prognostic factor for poor outcome for MMVD in small‐breed dogs. High serum Cys‐C concentrations were associated with a worse prognosis regardless of MMVD severity. Furthermore, serum Cys‐C could be a predictor of MMVD prognosis even in dogs with normal blood Cr.

## CONFLICT OF INTEREST DECLARATION

Validation data on measurement of canine serum cystatin C level was provided by FUJIFILM VET Systems Co, Ltd (Tokyo, Japan).

## OFF‐LABEL ANTIMICROBIAL DECLARATION

Authors declare no off‐label use of antimicrobials.

## INSTITUTIONAL ANIMAL CARE AND USE COMMITTEE (IACUC) OR OTHER APPROVAL DECLARATION

This study was approved by the ethics committee for animal clinical research of Gifu University (approval no. E22002). Informed consent by dog owners was waived because of the study's retrospective nature. All dog owners were given the option to opt out of the present study, which was conveyed via the bulletin board in Hashima Animal Hospital.

## HUMAN ETHICS APPROVAL DECLARATION

Authors declare human ethics approval was not needed for this study.
